# A novel 3-acyl isoquinolin-1(2*H*)-one induces G2 phase arrest, apoptosis and GSDME-dependent pyroptosis in breast cancer

**DOI:** 10.1371/journal.pone.0268060

**Published:** 2022-05-12

**Authors:** Lei Ma, Mengyao Bian, Hui Gao, Zhi Zhou, Wei Yi

**Affiliations:** Guangzhou Municipal and Guangdong Provincial Key Laboratory of Protein Modification and Degradation & Molecular Target and Clinical Pharmacology, The State Key Laboratory of Respiratory Disease, School of Pharmaceutical Sciences & The Fifth Affiliated Hospital, Guangzhou Medical University, Guangzhou, Guangdong, P. R. China; Southern Illinois University School of Medicine, UNITED STATES

## Abstract

Breast cancer is the most common malignancy among women worldwide, accordingly, numerous chemotherapeutic drugs have been discovered thus far. However, the development and application of these drugs is severely constrained because of their unclear mechanism. To address this issue, our previous work has defined 3-acyl isoquinolin-1(2*H*)-one derivatives as potent anti-tumor agents, among which the compound **4f** possessed relatively higher activity *in vitro*. In this study, we aim to further explore the anti-cancer effect and the underlying molecular mechanism of **4f** in breast cancer cells. Therefore, CCK8 assay was used to detect cell viability and flow cytometry was used to analyze cell cycle and apoptosis. Meanwhile, related proteins that regulate cell cycle and apoptosis were detected. The results showed that **4f** induced cell apoptosis and inhibited cell proliferation in breast cancer cells in a dose-depended manner without significant toxicity to human normal mammary epithelial cell. The cell cycle was arrested at G2 phase with the suppressed expression of the CDK1 protein. Additionally, **4f** was confirmed to induce the cell apoptosis with the up-regulation of bax, down-regulation of bcl-2, activation of cleaved-caspase3/7/9 and cleaved-PARP, together with the inhibition of MEK/ERK and p38 MAPK pathway. Moreover, the GSDME-mediated pyroptosis was also induced by **4f** in breast cancer cells. Together, these results demonstrated that **4f** could serve as a new and promising candidate for the treatment of breast cancer.

## Introduction

Breast cancer represents the most frequently diagnosed and the leading cause of cancer death in women worldwide [1]. In the last two decades, incidence and mortality rates of breast cancer have climbed sharply in China [2]. Additionally, previous studies revealed that one in eight women in the United States would develop breast cancer in her lifetime [3]. Modern treatment of breast cancer is multimodal, which includes surgery, radiation and chemotherapy. Nevertheless, drug resistance often limits the application of chemotherapy. Hence, it is vital to develop novel effective drug therapies against breast cancer.

Cancer cells are characterized by uncontrollable cell proliferation due to dysregulation of cell cycle regulation influenced by the deregulation of the activity of cell cycle related proteins [4, 5]. The cyclin E/CDK2 complex plays a critical role in G1/S transition, the cyclin A/CDK2 complex is required in the progression of S phase and the cyclin B1 and cdc2 complex (CDK1) is the specific regulator in G2/M phase transition and processes during mitosis [6–8]. Evasion of apoptosis is another hallmark of most types of cancer and the deregulation of apoptosis will leads to the uncontrolled growth of cancers [4]. Compared to apoptosis, the pyroptosis is a newly identified programmed cell death initiated by inflammatory caspases and numerous chemotherapeutic agents have been discovered to exert anti-tumor activities by inducing pyroptosis [9–11]. The gasdermin D (GSDMD) acts as the substrate of caspase 1/4/5/11 and can be cleaved to generate GSDMD-N fragment, which induces pyroptosis via forming the membrane pores [12, 13]. Alternatively, the gasdermin E (GSDME) gene, belongs to gasdermin superfamily, originally identified as deafness autosomal dominant 5 (DNFA5) [14], can function as a tumor suppressor gene in colorectal, gastric, melanoma, and breast carcinomas [15–17]. Shao *et al* reported that chemotherapy drugs could induce pyroptosis via the specific cleavage of GSDME by caspase 3 in the linker, thus generating a GSDME-N fragment that perforated membranes for pyroptosis induction [10]. Therefore, drugs that disrupt cancer cell cycle, restore apoptosis or pyroptosis might be effective against many types of cancers.

Demonstrated as important building blocks and promising skeletons, 3-acyl isoquinolin-1(2*H*)-ones and analogues exert anti-tumor effect in many cancers including glioblastoma, colorectal cancer and breast cancer [18–21]. The literature precedent disclosed that isoquinolinone derivatives had anti-tumor effect in breast cancer via inhibiting MAPK/ERK pathway [21, 22], which is a significant cell signaling pathway and involved in multiple cellular processes including cell proliferation and apoptosis. By virtue of an efficient and scalable rhodium(III)-catalyzed redox-neutral C-H annulation of *N*-methoxybenzamides with propargyl cycloalkanols, a series of 3-acyl isoquinolin-1(2*H*)-ones were synthesized for anti-tumor agents screening. Preliminary evaluation revealed that these motifs possessed anti-tumor effects equivalent to or even better than 5-FU in three different cancers *in vitro*, and delightfully, the compound **4f** exhibited the most potent anti-tumor activities against A549 and MCF-7 cell lines [23]. However, the anti-tumor mechanism of **4f** in breast cancer cells is unclear, which limits the further modification and development of such skeleton as the anti-tumor drug.

To address this drawback and in continuation of our interest in the development of drug therapy against cancer disease, we herein disclosed the effect of **4f** on cell proliferation, apoptosis and pyroptosis of breast cancer cells (MCF-7 and MDA-MB-213 cell lines) *in vitro*, as well as the underlying mechanisms. Our results demonstrated that **4f** induces G2 phase arrest of cell cycle, cell apoptosis and GSDME-mediated pyroptosis in breast cancer cells without significant toxicity to human normal mammary epithelial cell. Therefore, **4f** might serve as a lead compound for anti-tumor drug discovery.

## Materials and methods

### Chemicals and antibodies

The 3-acyl isoquinolin-1(2*H*)-one complex **4f** was synthesized according to a known procedure developed in our previous work. Cell counting kits-8 (CCK8) was purchased from Beyotime Biotechnology, dimethyl sulfoxide (DMSO) and RIPA lysis buffer were purchased from Sigma (Beverly, MA, USA). Primary antibodies such as anti-caspase3 antibody (cat#14220T), anti-cleaved-caspase9 antibody (cat#52873), anti-cleaved-caspase7 antibody (cat#8438), anti-parp antibody (cat#9542), anti-bcl-2 antibody (cat#15071T), anti-bax antibody (cat#5023), anti-β-tubulin antibody (cat#2128s), anti-ERK1/2 antibody (cat#4695T), anti-p-ERK1/2 antibody (cat#4370T), anti-MEK1/2 antibody (cat#9126s), anti-p-MEK1/2 antibody (cat#9154T), (HRP)-labeled anti-rabbit secondary antibody (Cat#7074) and (HRP)-labeled anti-mouse secondary antibody (Cat#7076) were purchased from Cell Signaling Technology. Anti-GSDME antibody (ab215191), anti-GSDMD antibody (ab210070), anti-p-MLKL antibody (ab187091) and anti-MLKL antibody (ab184718) were purchased from Abcam. Anti-CDK1 antibody (AF1516) was purchased from Beyotime Biotechnology.

### Cell culture

Human breast cancer cells MCF-7 and MDA-MB-231 were purchased from American Type Culture Collection (ATCC, Shanghai, China). Bother cells were cultured with Dulbecco’s Modified Eagle Medium cell culture medium (DMEM) (Gibco, USA) supplemented with 10% FBS (Gibco, USA), penicillin (100 μg/mL) and streptomycin (100 μg/mL, USA), all of them were cultured in 5% CO_2_ at 37°C in an incubator (Thermo Scientific, USA).

### CCK8 assay

Cells were plated in 96-well microtiter plates at a density of 5×10^3^/well and incubated in a humidified atmosphere with 5% CO_2_ at 37°C for 24 h. The tested compound was added into triplicate wells with different concentrations and 0.08% DMSO for the control. After they had been incubated for 24 h, 48 h or 72 h, respectively, 10 μL of CCK8 (Cell Counting Kit-8) solution was added into each well, and the plate was incubated for an additional 1 h. The absorbance (OD) was read on a microplate reader at 450 nm. The concentration causing 50% inhibition of cell growth (IC_50_) was determined by the Logit method using SPSS 13.0 software. All experiments were performed for three times.

### Colony formation assay

MCF-7 and MDA-MB-231 cells in the logarithmic growth phase were plated in a 6-well plate at a density of 500 cells per well. After 48 h, cells were incubated with DMSO (0.1%) or compound **4f** (1.25 μM, 2.5 μM, 5μM or 10 μM) for 72 h, and then were cultured in drug-free medium for 1 weeks. The cell colonies were fixed with 4% paraformaldehyde for 10 mins, stained with crystal violet for 20 mins, and then photographed. All experiments were performed for three times.

### EDU incorporation assay

Two stable MCF-7 and MDA-MB-231 cells (5×10^4^ cells/well) were plated in the 24-well plate. After treating with compound **4f** for 48 h, cells were treated with 50 μM EDU (Ribobio, China), which was obtained through adding 1μL EDU primary liquid into 1 ml DMEM medium with 10% FBS and 1% penicillin-streptomycin according to manufacturer’s instructions, for 2 h, and then fixed with 4% paraformaldehyde for 30 min at room temperature, incubated with 2 mg/ml glycine and 0.5% TritonX-100 for 5 min and 10 min, respectively; Then, cells were incubated with Apollo dye reagent (red) for 30 min at room temperature to react with EDU; Hoechst was used to stain the cell nucleus (blue). 10 random views at 200× magnification were obtained using inverted fluorescence microscope and the number of EDU positive cells was measured per field of view by IOD.

### Flow cytometry (FCM) analysis of cell cycle

After being cultured for 24 h, the MCF-7 and MDA-MB-231 cells were treated by different concentrations (0, 1.25, 2.5, 5, 10 μM) of compound **4f** for 24 h. Afterwards, the cells were washed and resuspended in cold PBS and incubated in ice-cold 75% ethanol for 12 hours. The cells were then centrifuged at 1000 rpms for 10 mins and washed using cold PBS for 2 times, then resuspended in propidium iodide (PI) master mix (40 μg/ml PI and 100 μg/ml RNase A in PBS) at a density of 5×10^5^ cells/ml and incubated at 37°C for 30 mins before analysis with flow cytometry (Beckman Coulter, USA) as described. The excitation and emission wavelengths of PI were 535/615 nm. The cell cycle phase analysis was performed by FlowJo 7.6 software.

### Flow cytometry (FCM) analysis of cell apoptosis

Apoptotic cell death was determined by flow cytometry analysis using Annexin V-FITC and propidium iodide (PI) assay kit (BD, PharMingen, San Diego, CA). After being cultured for 24 h, the cells were treated by different concentrations (0, 1.25, 2.5, 5, 10 μM) of compound **4f** for 48 h. MCF-7 and MDA-MB-231 cells were collected, washed with cold PBS, suspended in 5 μL of Annexin V binding buffer and stained with 5 μL of PI. The cells were mixed gently, incubated in the dark for 20 mins, and washed. The samples were analyzed with a FACS (Beckman Coulter, USA).

### Quantitative real-time PCR

The total RNA of cells was extracted using TRIZOL reagent and purified by 75% ethyl alcohol. Complementary DNA was synthesized using iscript reverse transcription kit (Takara) according to manufacturer’s instructions. Quantitative real-time PCR was performed with SYBR Green Premix reagent (Takara) on the 7300 real-time PCR system (ABI). Two-step PCR amplification standard procedure was used according to manufacturer’s instructions. The first stage: PCR was initiated at 95°C for 30 seconds to hot-start DNA polymerase and denature the template. The second stage (PCR reaction): 40 cycles that consisted of 95°C for 5 seconds, 60°C for 31 seconds. Data were calculated using the 2-ΔΔCt method. The primers used in the quantitative real-time PCR were synthesized in Life Technologies (S2 Table).

### LDH release assay

LDH release was measured using LDH Cytotoxicity Assay Kit (Beyotime Biotechnology) according to the manufacturer’s instructions. The absorbance value at 490 nm was then measured. Each experiment was repeated for three times.

### Western blotting analysis

Equal amounts of cellular proteins were loaded onto SDS-polyacrylamide (Bio-Rad), electrophoresed, and transferred onto PVDF membranes (Bio-Rad) by wet electrophoretic transfer. The membranes were then blocked for 1 h at room temperature with 5% non-fat milk in TBST, and incubated with primary antibodies overnight at 4°C, followed by horseradish peroxidase-linked secondary antibody (Cell Signaling Technology) for 1 h at room temperature. The proteins were detected by using an enhanced chemiluminescence (ECL) substrate (Thermo), according to the manufacturer’s instructions. The β-tubulin was used as an internal control.

### Measurement of mitochondrial membrance potential

The fluorescent dye JC-1 (Beyotime Institute of Biotechnology, China) was applied to evaluate the mitochondrial membrance potential (Δψm). Briefly, MCF-7 and MDA-MB-231 cells were seeded in the 24-well plates at a density of 1×10^4^ cells/well for 24 h, followed by the treatment with different concentrations (0, 1.25, 2.5, 5, 10 μM) of compound **4f** for 48 h. Then the cells were incubated with 0.5 mL JC-1 staining working solution for 20 min at 37°C. The cells were further washed twice with JC-1 staining buffer, then the fluorescence of monometric (green) or aggregate JC-1 (red) was examined at an emission wavelength of 525 or 597 nm, respectively, under a beckman flow cytometer. The assay was repeated in triplicate.

### Statistical analyses

All data were analyzed with the unpaired study t-test by using GraphPad 5, and the data were presented as the mean ± SEM. All cell culture experiments were performed independently for at least three times and in triplicate each time. P value of <0.05 or <0.001 was considered statistically significant

## Results

### Effects of compound 4f on cancer cell viability

**Fig 1:**
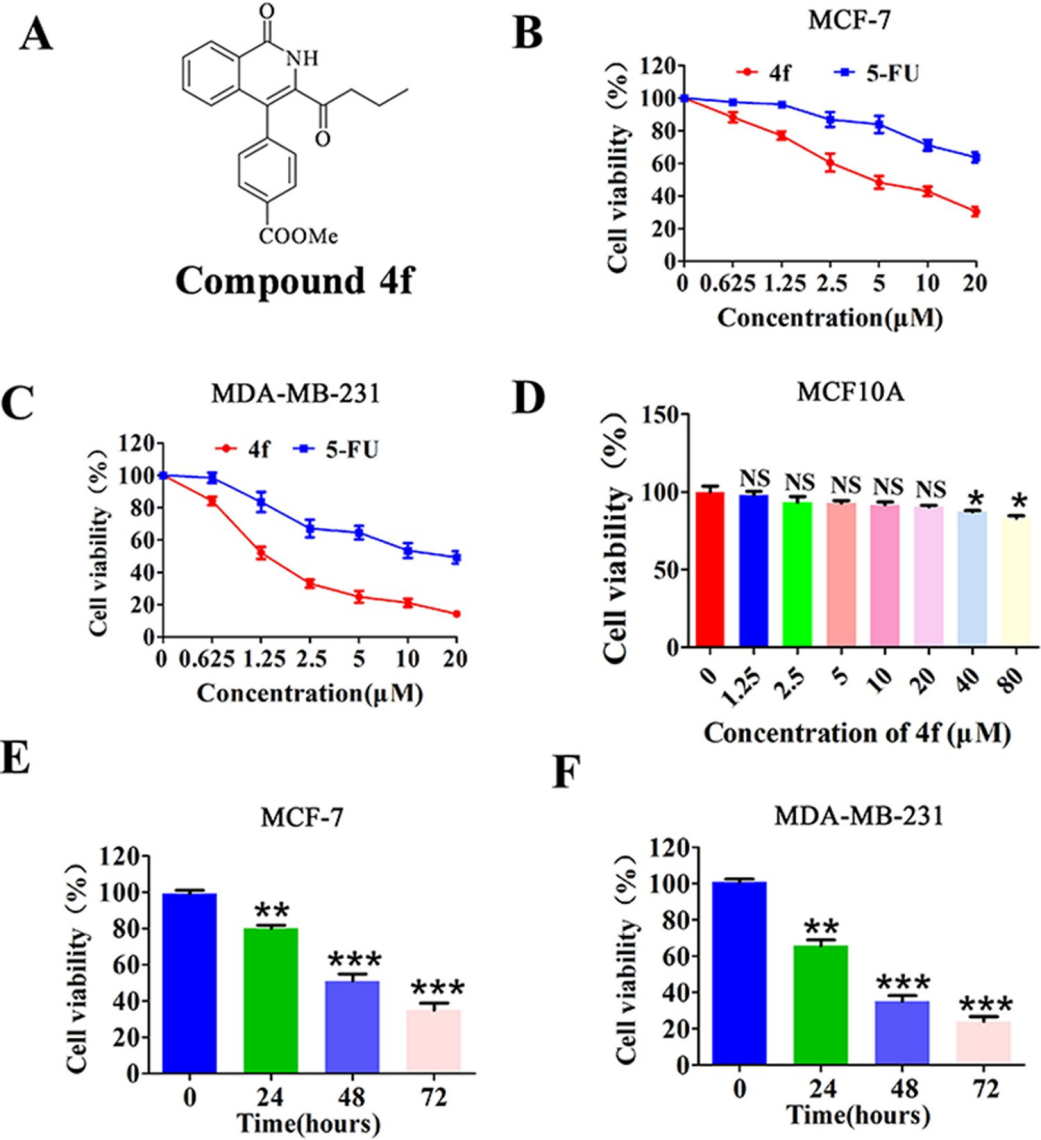
Compound 4f inhibits breast cancer cell viability. (A) The structure of compound **4f**. (B-D) The effect of compound **4f** on the viability of the breast cancer cells MCF-7 and MDA-MB-231 and human normal mammary epithelial cell MCF10A by CCK8 assay at the indicated concentration, n = 3. (E, F) Time-dependent of **4f** on the viability of the MCF-7 and MDA-MB-231 cells assessed by CCK8 assay. NS, not significant different from 0 group, *significantly different from 0 group. *p<0.05, **p<0.01 and ***p<0.001, n = 3.

The efficient synthesis of diverse 3-acyl isoquinolin-1(2*H*)-ones and the preliminary evaluation of the anti-tumor activity against several human cancer cells (*e*.*g*. HepG2, A549, MCF-7 and SH-SY5Y) have been developed in our previous work, among which the compound **4f** (Fig 1A) showed excellent anti-tumor effect in MCF-7 cells. Encouraged by this result, further examination of the anti-tumor activity of **4f** against breast cancer cells including MCF-7 and MDA-MB-213 was next conducted. As shown in Fig 1B and 1C, **4f** showed relatively higher anti-tumor activity than that of 5-FU in a dose-dependent manner determined by CCK8 assay in both breast cancer cells, with the indicated half-maximal inhibitory concentration (IC_50_) ranging from 2.39±0.63 μM to 5.65±0.57 μM (S2 Table). Further evaluation on the cytotoxicity of compound **4f** against human normal mammary epithelial cell MCF10A demonstrated the selective killing effect of **4f** on cancer cells (Fig 1D). Subsequently, the treatment of MCF-7 and MDA-MB-213 cells with **4f** for 24, 48 and 72 h, respectively, indicated that this compound remarkably reduced cell viability in a time-dependent manner (Fig 1E and 1F).

### Compound 4f inhibits cell proliferation and induces cell cycle arrest in breast cancer

**Fig 2:**
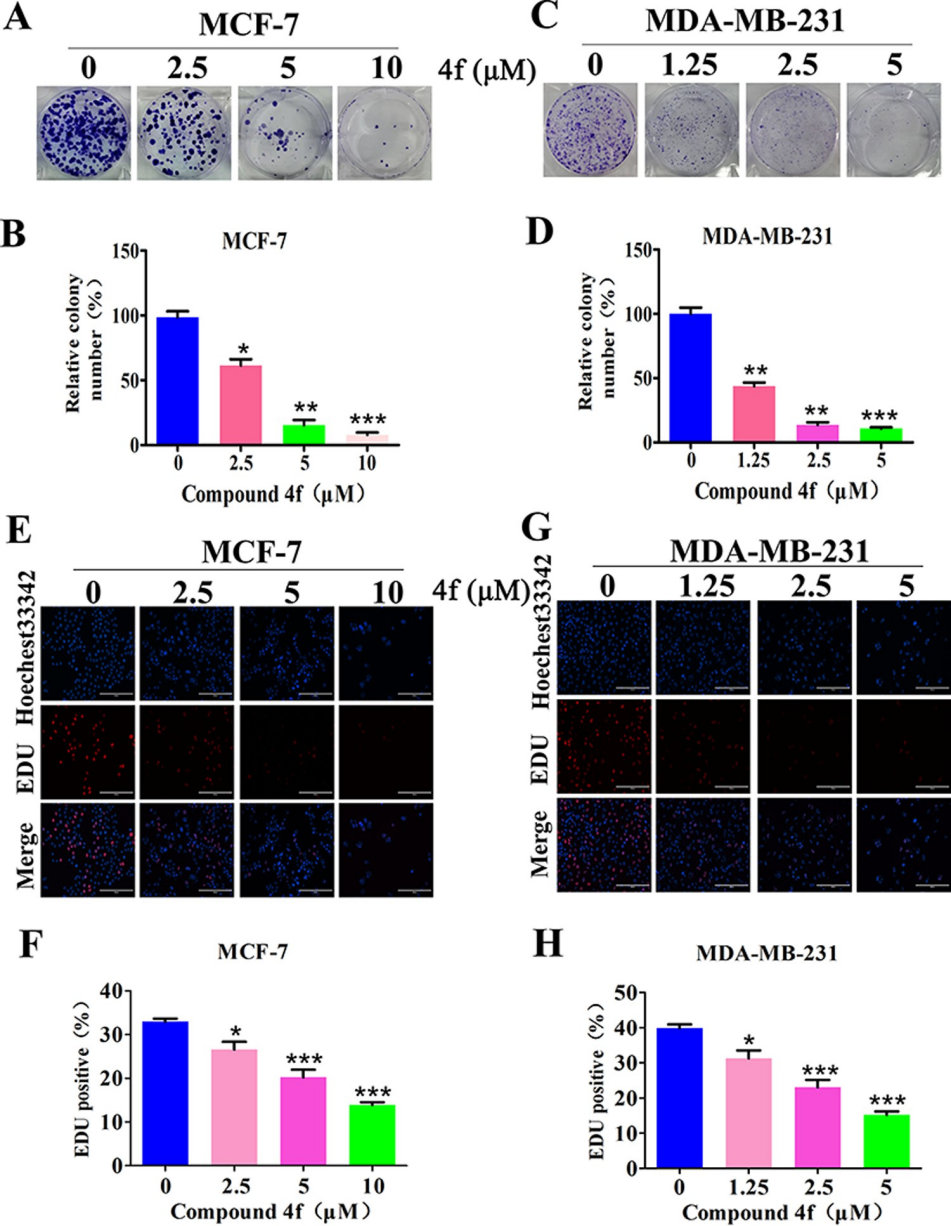
Compound 4f inhibits cells proliferation in MCF-7 and MDA-MB-231 cell lines. (A-D) The clonogenic assay and the number of colonies formation analyses. (E-H) The EDU incorporation assay on MCF-7 and MDA-MB-231 cells after treated with compound **4f** for 48 h. The red fluorescent staining (EDU-positive) and blue fluorescent staining (Hoechst 33258 staining) cells represent the proliferating and total cells, respectively (E, G). Quantification of EDU-incorporating MCF-7 and MDA-MB-231 cells (F, H). *significantly different from 0 group.*p<0.05, **p<0.01 and ***p<0.001, n = 3.

To gain more insight into the inhibitory effects of isoquinolinone **4f** on cancer cell proliferation, we next conducted the colony formation assays. The results clearly showed that clone formation of the two tumor cell lines was reduced in a concentration-dependent manner after exposure to **4f** (Fig 2A–2D). In addition, the EDU incorporation assay was performed to investigate the anti-proliferative effects of **4f** against MCF-7 (Fig 2E and 2F) and MDA-MB-213 cells (Fig 2G and 2H). To the end, the percentages of EDU positive cells decreased with the increasing of the concentration of **4f**, indicating that the proliferation of both breast cancer cells was strongly inhibited by compound **4f**, which was consistent with the cell viability data.

**Fig 3:**
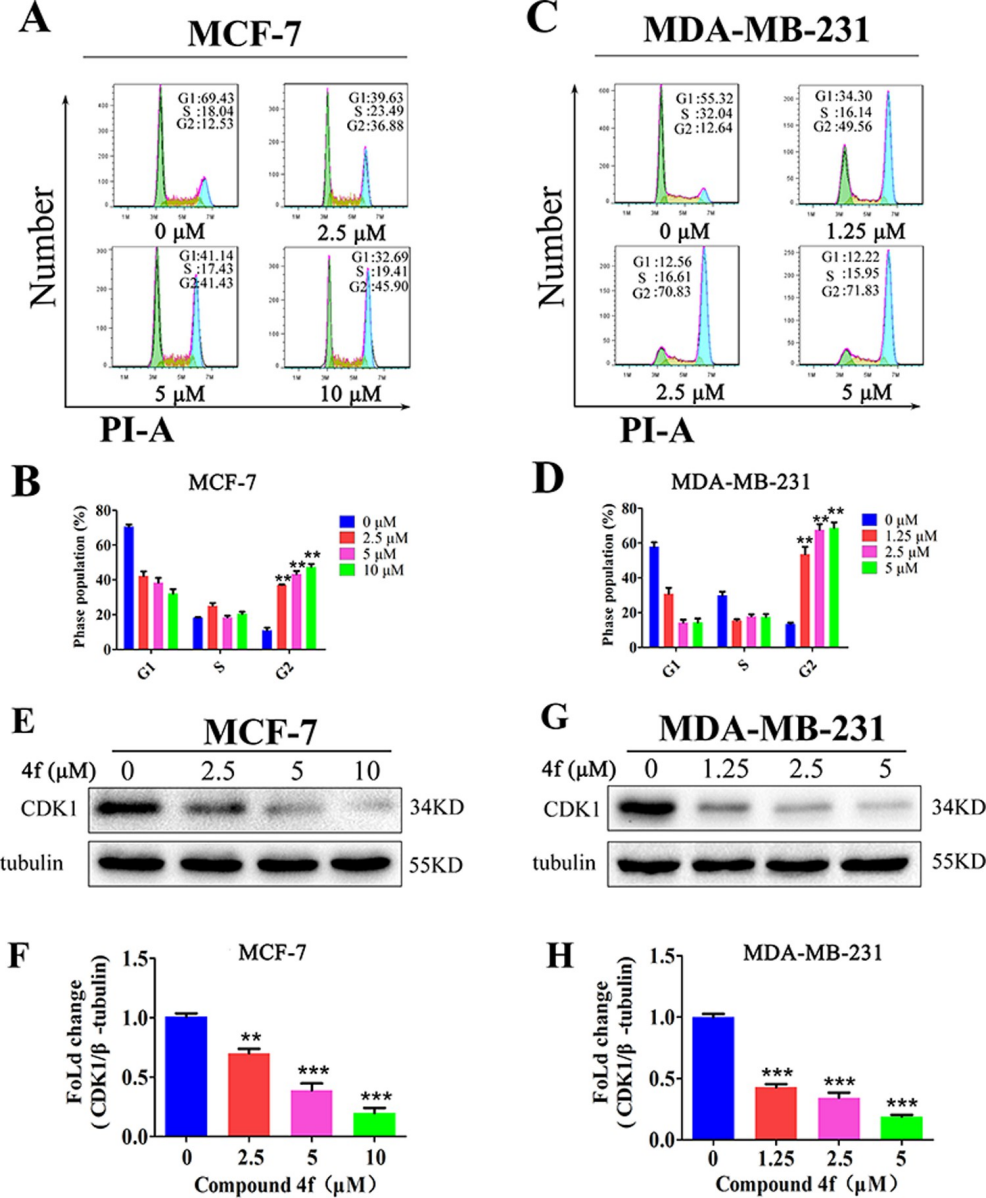
Induction of cell cycle arrest at the G2 phase by compound 4f in breast cancer cells. (A-D) Compound **4f** increased the proportion of cells in G2 phase of the cell cycle. (E-H) Western blotting assay for the analysis of CDK1 expression in **4f**-treated MCF-7 and MDA-MB-231 cells at indicated concentration for 24 h. *significantly different from 0 group. **p<0.01 and ***p<0.001, n = 3.

Subsequently, flow cytometry analysis of **4f**-treated breast cancer cell lines was carried out to further determine whether **4f** inhibited the cell growth by inducing cell cycle arrest. The results indicated that the proportions of MCF-7 and MDA-MB-231 cells were significantly increased in G2 phase in a dose-depended manner treated by **4f** (Fig 3A–3D). Meanwhile, western blotting analysis further confirmed that the compound **4f** induced cell cycle arrest in G2 phase in MCF-7 and MDA-MB-231 cells due to the reduction of the protein level expression of cyclin-dependent kinase 1 (CDK1), which represents an important regulator that control cells from G2 phase into M phase [8] (Fig 3E–3H).

### Compound 4f promotes breast cancer cells apoptosis

**Fig 4:**
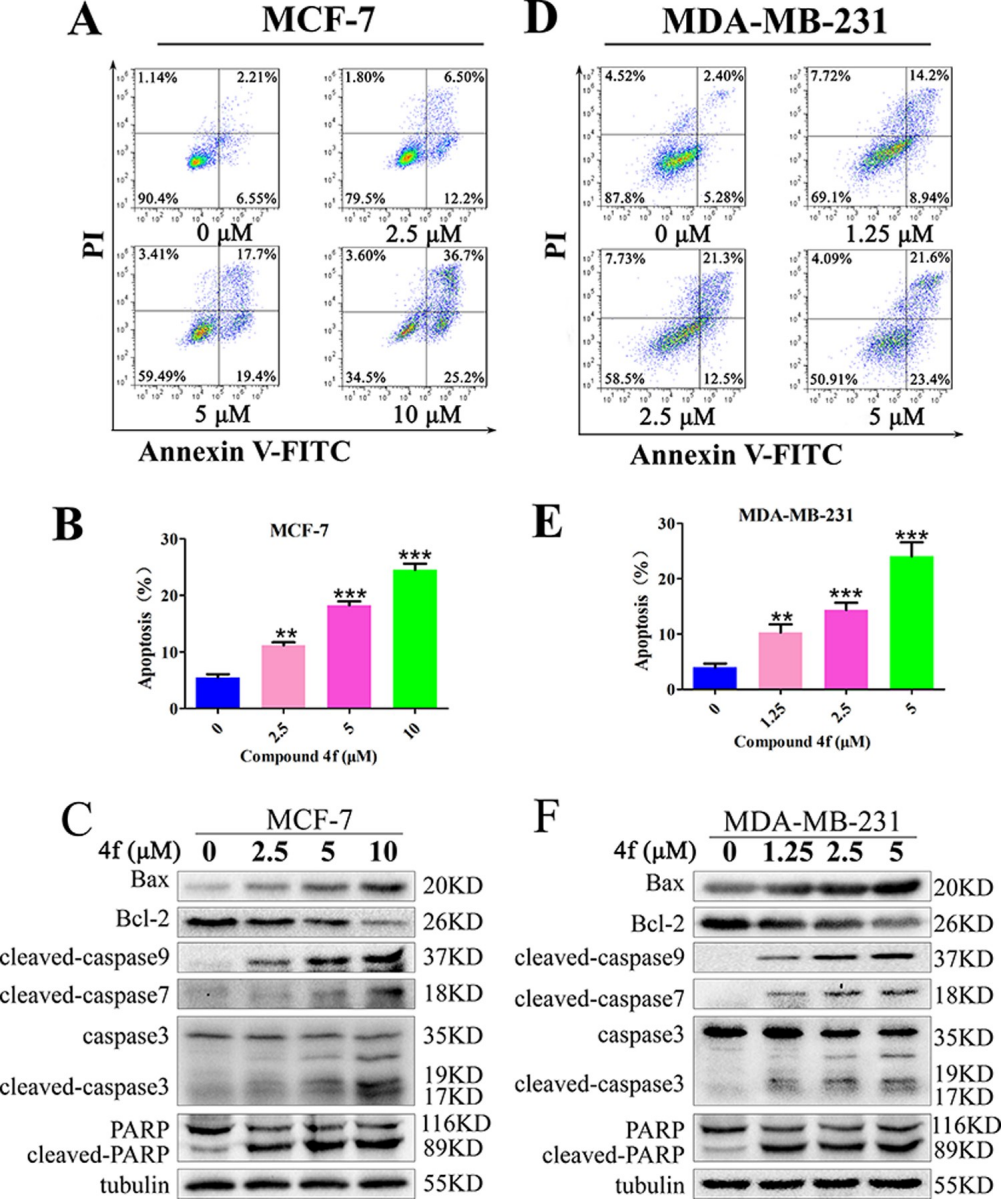
Compound 4f induced apoptosis in MCF-7 and MDA-MB-231 cells. Annexin V-FITC and Propidium Iodide (PI) assay kit were used to detect cell apoptosis, which was analyzed by flow cytometry after the treatment with **4f** for 48 h at different concentrations (0, 1.25, 2.5, 5, 10 μM) in MCF-7 (A, B) and MDA-MB-231 (D, E) cells. Western blotting analysis of the expression of apoptosis related proteins in MCF-7 (C) and MDA-MB-231 (F) cells after the treatment with **4f** for 24 h. *significantly different from 0 group. **p<0.01 and ***p<0.001, n = 3.

Except for the cell cycle arrest, cell apoptosis is also responsible for the anti-proliferation activity of chemotherapy drugs [24]. Consequently, the ability of isoquinolinone **4f** on inducing apoptosis of MCF-7 and MDA-MB-231 cells was next explored to further probe the possible molecular mechanism for suppressing the breast cancer cells viability. Annexin V-FITC and Propidium Iodide (PI) assay kit were used to detect the cell apoptosis, which was analyzed by flow cytometry. The results displayed that **4f** induced marked cell apoptosis in a concentration-depended manner, with the percentage of apoptotic cells ranged from 6.55% to 25.2% and 5.28% to 23.2% in MCF-7 (Fig 4A and 4B) and MDA-MB-231 cells (Fig 4D and 4E), respectively. Since mitochondria-mediated intrinsic and cell death receptor-mediated extrinsic paths represent the mainly two types of apoptosis pathway, a series of western blotting assay was next conducted to distinguish the apoptosis pathway induced by compound **4f** in breast cancer cells. The results revealed that **4f** had no effect on the cleavage of caspase 8, which is an important regulation factor for the extrinsic pathway. In sharp contrast, the compound **4f** significantly up-regulated the pro-apoptotic protein Bax expression while reduced anti-apoptotic protein Bcl-2 expression, both of which as key factors to regulate the process of intrinsic apoptosis. Besides, the protein expression level of cleaved-PARP, cleaved-caspase 3, cleaved-caspase 7 and cleaved-caspase 9 in the breast cancer cells increased after treated with compound **4f** (Fig 4C and 4F). Together, these results clearly demonstrated that the mitochondria-mediated intrinsic apoptosis pathway should be involved.

**Fig 5:**
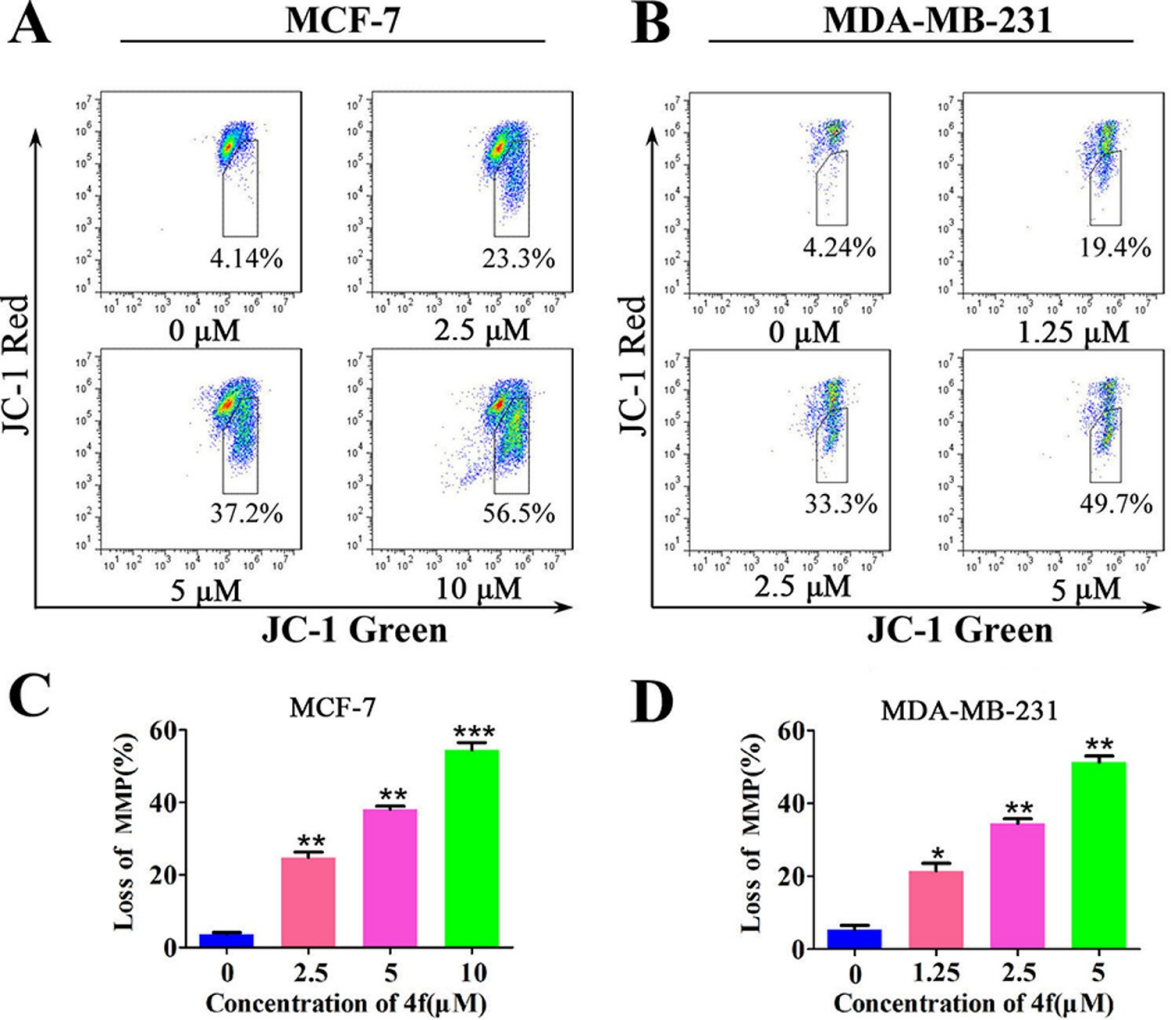
Compound 4f induces reduction of the MMP in breast cancer cells. Cells were treated with **4f** at a concentration of 0 μM, 2.5 μM, 5 μM, 10 μM in MCF-7 cell line (A) and 0 μM, 1.25 μM, 2.5 μM, 5 μM in MDA-MB-231 cell line (B) for 24 h, the MMP was measured by JC-1. (C, D) The ratio of decreased MMP. *significantly different from 0 group. *p<0.05, **p<0.01 and ***p<0.001, n = 3.

On the other hand, the decrease of mitochondrial membrane potential (MMP) is a key indicator of early apoptosis, which can cause the release of apoptotic factors and activate the downstream apoptotic pathways [25]. Consequently, the MMP was further analyzed in MCF-7 and MDA-MB-231 cells by flow cytometry using a JC-1 MMP Kit, which acts as a fluorescence probe and selectively enters into the mitochondria and reversibly change color from red (the aggregate form) to green (the monomeric form) when mitochondrial membrane potential declines [26]. As shown in Fig 5A–5D, the MMP reduced significantly after treated with compound **4f** for 24 h in MCF-7 and MDA-MB-231 cells.

### Compound 4f inhibits MEK/ERK and p38 MAPK pathways in breast cancer cells

**Fig 6:**
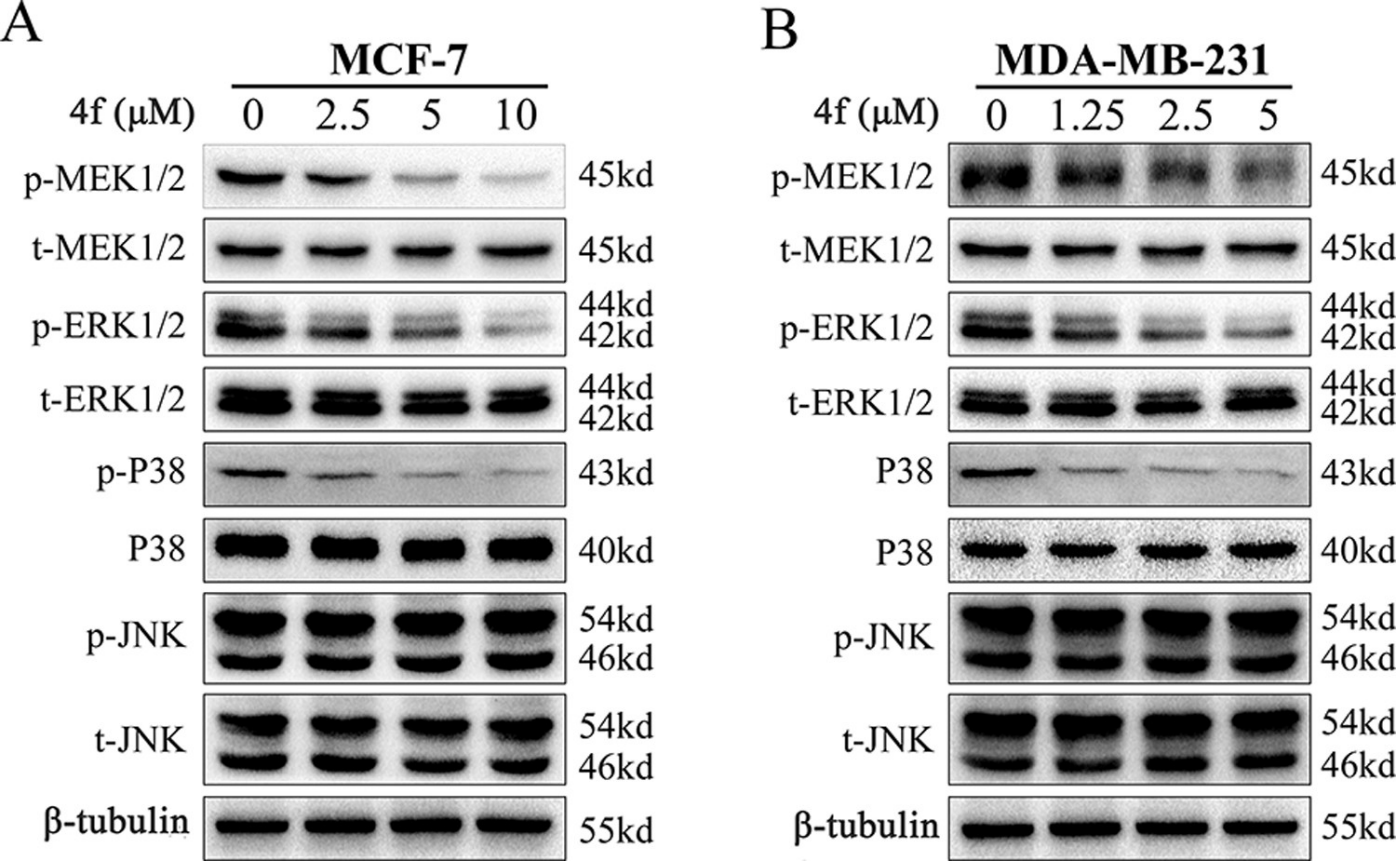
4f inhibits MEK/ERK and p38 MAPK pathways in breast cancer cells. Cells were treated with **4f** at a concentration of 0 μM, 2.5 μM, 5 μM, 10 μM in MCF-7 cell line (A) and a concentration of 0 μM, 1.25 μM, 2.5 μM, 5 μM in MDA-MB-231 cell line (B) for 24 h, the expression of phosphorlated and total ERK1/2, p38, JNK and MEK1/2 were detected by western blotting assays. All the data repeated for three times.

Numerous studies have reported that the induction of cell apoptosis is associate with MAPK pathway [24, 27, 28], among which the isoquinolinone derivatives were proved to present the anti-tumor effect in breast cancer via inhibiting MAPK/ERK pathway [21, 22]. Thus, the protein expression levels in MAPK pathway were detected in breast cancer cells to determine whether **4f** affects the MAPK pathway by western blotting assays. As listed in Fig 6, the total protein expression of ERK1/2, p38 and JNK were not altered under the treatment of **4f**, while the phosphorylation of ERK1/2 and p38 were reduced with the expression of phosphorylated JNK remained unchanged. ERK1/2 represents a serine/threonine kinase that positively regulated by phosphorylation and mediated by MEK1/2 [29]. The examination of the MEK1/2 protein revealed that **4f** could reduce the phosphorylation of MEK1/2 without altering the total protein expression. These results confirmed that **4f** inhibited MEK/ERK and p38 MAPK pathways in breast cancer cells.

### Compound 4f induces cell pyroptosis by caspase 3 mediated cleavaged of GSDME

Pyroptosis, a newly identified programmed cell death executed by gasdermins [12], is essentially different from apoptosis, a non-inflammatory form of programmed cell death. Precedented studies prove that pyroptosis plays a significant role in the treatment of tumors with chemotherapy drugs. Classical chemotherapy drugs including doxorubicin, peclitaxel, cisplatin, lobaplatin and 5-FU can induce pyroptosis effectively via caspase 3/GSDME in the treatment of tumors [9, 15, 16, 30–32]. Moreover, diverse bioactive molecules including chalcones, thiopyran derivatives and piperlongumine analogues have also been proven to induce pyroptosis [33–35], indicating that pyroptosis provides a novel therapeutic strategy for tumors. Considering the above observation that isoquinolinone **4f** had an effect on the cleavage of caspase 3, we presumed that **4f** might induce pyroptosis in breast cancer cells.

**Fig 7:**
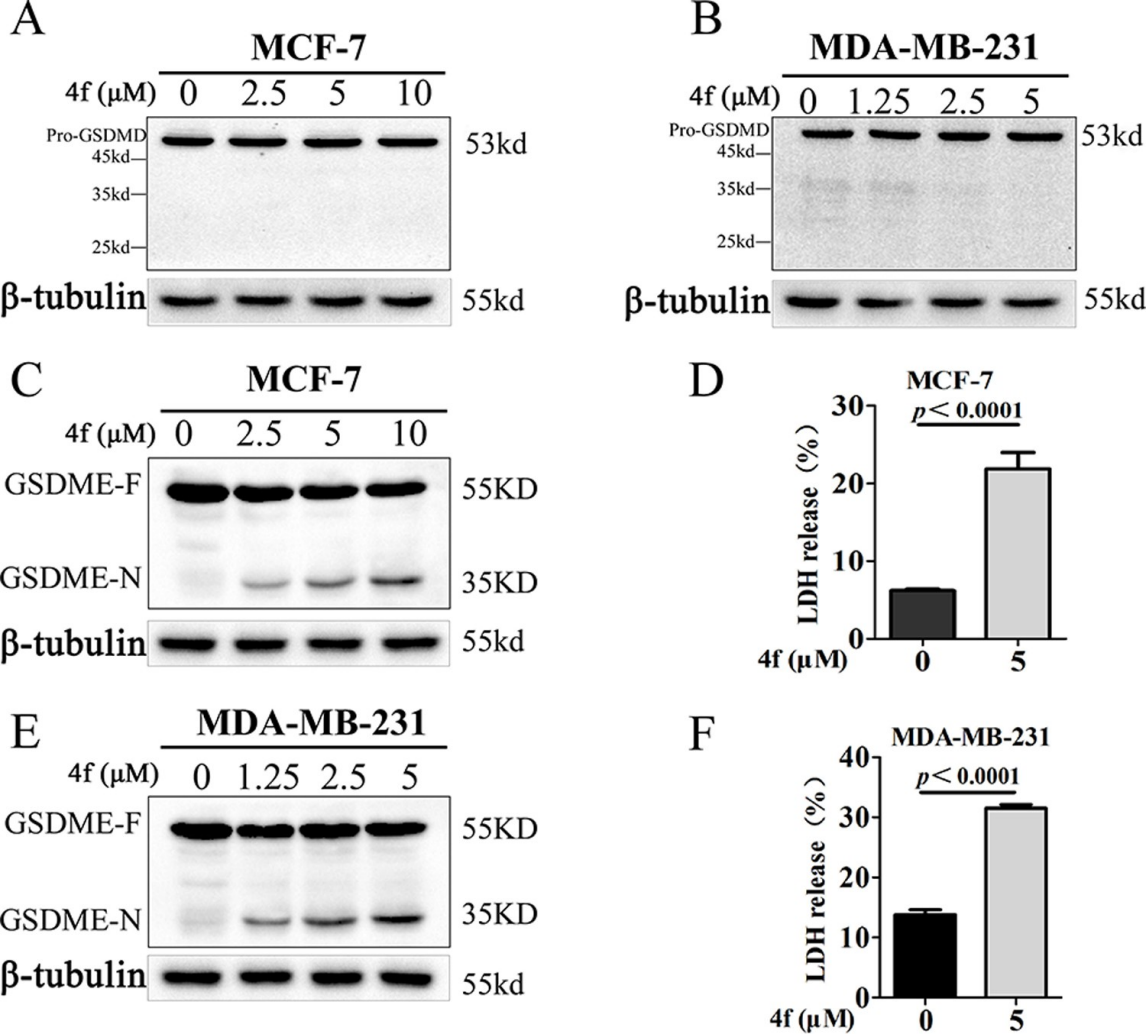
GSDME rather than GSDMD is cleaved during cell pyroptosis of breast cancer cells induced by 4f. (A, B) Full-length GSDMD (GSDMD-F) and GSDMD-C terminal (GSDMD-C) were detected by western blotting in MCF-7 and MDA-MB-231 cells treated with different doses of **4f**. (C, E) Full-length GSDME (GSDME-F) and GSDME-N terminal (GSDME-N) were analyzed by western blotting in MCF-7 and MDA-MB-231 cells treated with different doses of **4f**. (D, F) LDH release of MCF-7 and MDA-MB-231 cells treated with **4f** at the concentration of 0 or 5 μM. All the data repeated for three times.

The GSDMD-N fragment generated by the cleavage of GSDMD via activating caspase 1/4/5/11 derives pyroptosis through its pore-forming activity [36–39]. Alternatively, GSDME-dependent cell death is another form of pyroptosis, which induced by TNF-α or chemotherapy drugs [11]. Recent studies reported that GSDME was specifically cleaved by caspase 3 in the linker, generating a GSDME-N fragment that perforated membranes for pyroptosis induction [10, 40]. To gain more insight into the effect of compound **4f** on pyroptosis induction and clarify the pyroptosis pathway, a set of western blotting was conducted and the results showed that no cleavage of GSDMD occurred in MCF-7 and MDA-MB-231 cells treated by **4f** (Fig 7A and 7B). Nevertheless, the GSDME cleavage was induced and increased by **4f** in both breast cancer cell lines in a dose-depended manner (Fig 7C and 7E). In addition, the release of proinflammatory factor, lactate dehydrogenase (LDH), was significantly elevated compared with the control group, demonstrating the plasma membrane rupture and leakage under the treatment of **4f** (Fig 7D and 7F). Moreover, MCF-7 and MDA-MB-231 cells were respectively treated with **4f** at the concentration of 5 μM and 2.5 μM for 24 h, and then, the morphology of cells were photographed with microscope (S1 Fig). The cells that marked with red arrow exhibited large bubbles emerging from the plasma membrane and cell swelling, which are represent cells undergo pyroptosis. As the morphology and Annexin-V/PI character of necroptosis and pyroptosis are very similar, we also evaluated whether **4f** induced necroptosis. p-MLKL is the key protein regulated necroptosis, for this concern, we detected the phosphorylation of MLKL signal that regulate necroptosis by western blotting analysis using the antibodies against MLKL and p-MLKL, necrosis inducer, a combination of TNF-a (T, 20 ng/ml), Smac mimetic (S, 100 nM), and a pan-caspase inhibitor z-VAD-FMK (Z, 20 μM) was used as positive control. The results indicated that no phos-MLKL signal was observed after treating with **4f** (S2 Fig), suggesting that **4f** did not induce necroptosis in MCF-7 and MDA-MB-231 cells after treating with **4f**. Taken together, these results suggested that **4f** could induce pyroptosis through the cleavage of GSDME rather than GSDMD.

**Fig 8:**
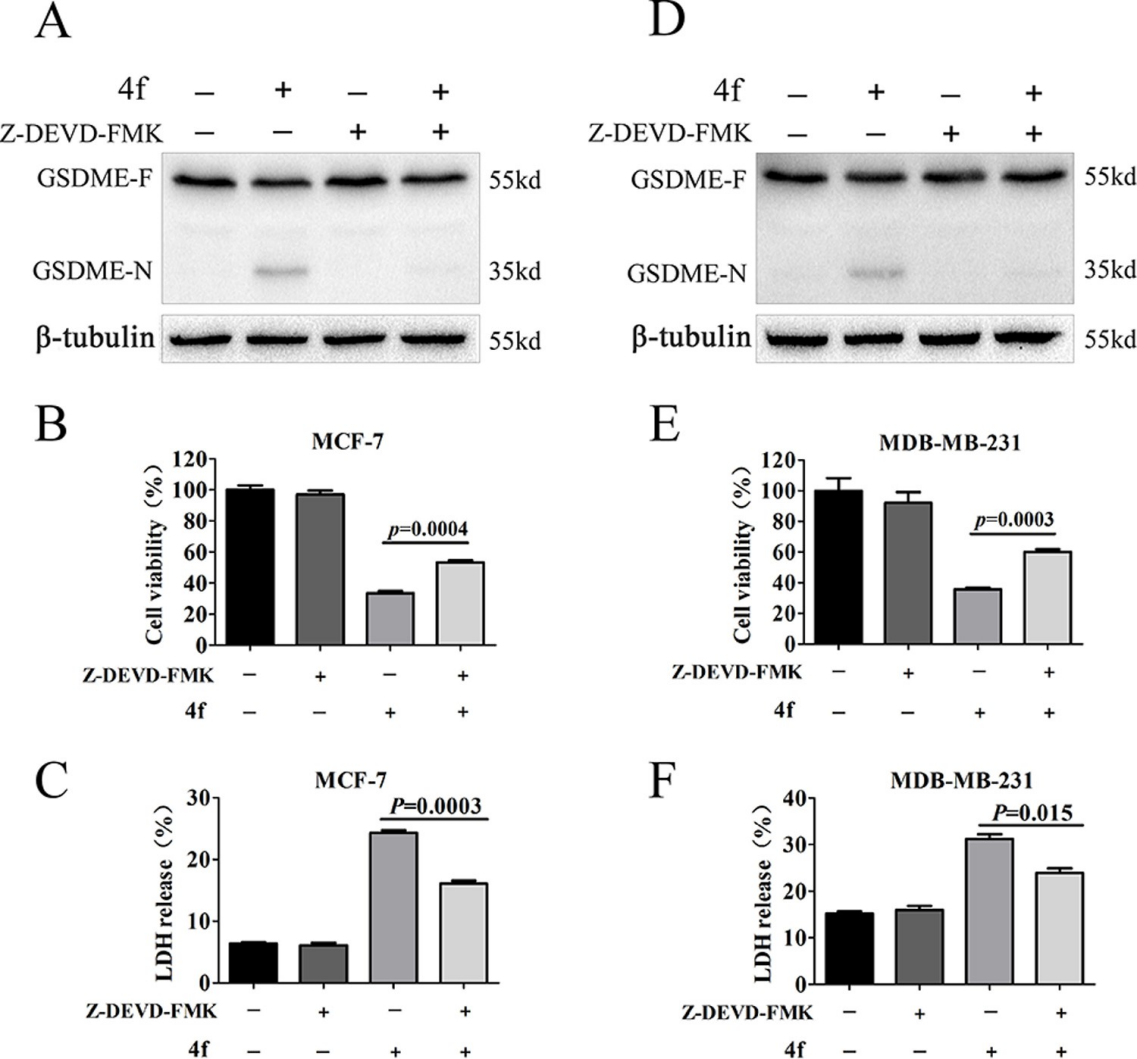
z-DEVD-FMK reduces the cleavage of GSDME. (A, D) Western blotting analysis of full-length GSDME (GSDME-F) and GSDME-N terminal (GSDME-N) in MCF-7 and MDA-MB-231 cells treated by **4f** (5 μM) in the presence or absence of z-DEVD-FMK (50 μM). (B, E) Cell viability analysis by CCK8 kit. (C, F) LDH release was measured using LDH cytotoxicity assay kit. All the data repeated for three times.

To verify the involvement of caspase 3 in **4f**-triggered pyroptosis, the MCF-7 and MDA-MB-231 cells were pre-treated with caspase 3 specific inhibitor z-DEVD-FMK for 2 h and then treated with **4f** for 24 h. The results revealed that z-DEVD-FMK could inhibit the cleavage of GSDME (Fig 8A and 8D) and reduce the release of LDH (Fig 8C and 8F) triggered by **4f**. Moreover, CCK8 assay showed that z-DEVD-FMK reversed the cell viability induced by **4f** (Fig 8B and 8E). These results demonstrated that the cleavage of GSDME by caspase 3 was responsible for **4f**-induced pyroptosis in MCF-7 and MDA-MB-231 cells.

## Discussion

Recent studies found that a number of isoquinolinone derivatives exhibited anti-tumor effect in many cancers by inducing cell cycle arrest and apoptosis [19, 20], and a series of 3-acyl isoquinolin-1(2*H*)-ones was obtained and identified as anti-tumor agents against four different cancer cell lines in our previous study [23], among which compound **4f** showed potent anti-tumor effect in MCF-7 cell line. In this study, we demonstrate that **4f** reduces cell viability in MCF-7 and MDA-MB-231 cell lines by CCK8 assay. Further investigation on cell proliferation indicates that **4f** reduces the number of EDU positive cells and suppresses the colony formation in MCF-7 and MDA-MB-231 cell lines, and the results from flow cytometry demonstrate that **4f** induces cell cycle arrest in G2 phase. Cyclin-dependent kinase 1 (CDK1), a complex of cyclin B1 and cdc2, is an important regulator that control cells from G2 phase into M phase [8]. Indeed, the reduction of CDK1 protein expression is observed in MCF-7 and MDA-MB-231 cells after treating with **4f**, which is responsible for cell cycle arrest. These results demonstrate that **4f** inhibits cell proliferation by inducing cell cycle arrest in G2 phase.

We further investigate whether **4f** could induce apoptosis in breast cancer cells. The results showed that **4f** induced marked cell apoptosis in a dose-depended manner in MCF-7 and MDA-MB-231 cells. In addition, **4f** promoted the expression of cleaved-PARP, cleaved-caspase3, cleaved-caspase7, and cleaved-caspase9, as well as up-regulated the protein level of Bax while decreasing the protein level of Bcl-2, which are the key proteins that regulate intrinsic apoptosis pathway. Additionally, the results from flow cytometry showed that **4f** could decrease the mitochondrial membrane potential, a feature of early apoptosis. Taken together, our data indicate that **4f** promotes cell apoptosis via mitochondria pathway in breast cancer cells. Isoquinolinone derivatives have been reported to have anti-tumor effect in breast cancer via inhibiting MAPK/ERK pathway [21]. Thus, the changes in MAPK pathway in breast cancer cells after treating with **4f** were analyzed by western blotting assays. In our study, we discovered that **4f** inhibited the expression of phosphorylation of p38 and ERK1/2, as well as the phosphorylation of MEK1/2, which can positively regulate ERK1/2. These results confirmed that **4f** inhibited MEK/ERK and p38MAPK pathways, which might be another reason for promoting cell apoptosis.

Pyroptosis, a newly identified programmed cell death [12], which is essentially different from apoptosis. Recent advances have shown all Gasdermin family members except for a shortened one, DFNB59, can mediate pyroptosis. And GSDME-N is specifically cleaved by caspase3 in the linker, generating GSDME-N fragment, which can induce pyroptosis [10, 40]. Recent studies have reported that pyroptosis plays a significant role in the treatment of tumors with chemotherapy drugs. Besides, the newly synthesized compounds including chalcone analogues, a thiopyran derivative, a piperlongumine analogue can also induce pyroptosis [38–40], indicating that pyroptosis provides a novel therapeutic strategy for tumors. The results in our study indicated that **4f** promoted the release of LDH and induces the cleavage of GSDME while fail to promote the cleavage of GSDMD. What’s more, the cleavage of GSDME and the release of LDH could be inhibited by Z-DEVD-FMK, a specific inhibitor of caspase3, meanwhile, cell viability is restored in **4f** treated breast cancer cells after treating with Z-DEVD-FMK. These results indicated that **4f** induced pyroptosis through caspase3/GSDME pathway. As caspase3 is also a core protein in regulating apoptosis, the correlation between apoptosis and pyroptosis in breast cancer cells treated with **4f** need to be further explored.

In summary, having developed the efficient synthetic approach of diverse 3-acyl isoquinolin-1(2*H*)-ones and preliminarily explored their bioactivity as anti-tumor agents, we herein further evaluated the pharmacodynamics of the most active compound **4f** *in vitro* against the breast cancer cell lines including MCF-7 and MDA-MB-231 cells. The results demonstrated that **4f** inhibited cell proliferation by inducing cell cycle arrest in G2 phase and promoted cell apoptosis via mitochondria-mediated intrinsic apoptosis pathway. Meanwhile, the MEK/ERK and p38 MAPK pathways were also inhibited by **4f**. Additionally, the pyroptosis induction triggered by **4f** was also confirmed and the caspase 3/GSDME pathway was verified. Taken together, these results suggested that the isoquinolinone complex **4f** may serve as a lead compound for anti-tumor drug discovery against breast cancer, and more importantly, it also provide a mechanistic basis for employing such isoquinolinone skeleton as the anti-cancer discovery and development. Having these solid data in hand, further investigation on targeted structural modifications together with the comprehensive *in vivo* studies of **4f** is in progress.

## Supporting information

S1 Fig The morphology of cells after the treatment of compound 4f. Representative microscopic images of MCF-7 and MDA-MB-231 cells after treated with **4f** for 24 h with cell swelling. Red arrowheads indicate the characteristic balloon in the cell membrane, which are represent cells undergo pyroptosis.
(TIF)

S2 Fig The influence of compound 4f on the key protein that regulate necroptosis. MCF-7 and MDA-MB-231 cells were treated with **4f** or TSZ for 24 h, the total protein was extracted respectively, and then, the protein expression of p-MLKL and MLKL were analysized by western blotting analysis. TSZ is a combination of TNF-a (T, 20 ng/ml), Smac mimetic (S, 100 nM), and a pan-caspase inhibitor z-VAD-FMK (Z, 20 μM), which is used as necrosis inducer.
(TIF)

S1 Table Primer sequences used for qPCR. (DOCX)

S2 Table The IC_50_ of the synthesized compound 4f on both huaman breast cancer cell lines. Cells were treated with different concentrations of compound **4f** for 48 h. IC_50_ values are the mean±SD (n = 3).
(DOC)

S1 File Supporting information for HPLC characterization data of 4f. HPLC conditions: Thermo Fisher C_18_ column HYPERSIL GOLD ODS, 250 x 4.6 mm I.D, S-5 μm (30:70 MeCN: H_2_O, 1.0 mL/min, 30°C, 254 nm); tr = 20.2 min, 99.3%. Instrument model: C_18_ chromatographic column HYPERSIL GOLD ODS, 250×4.6 mm I.D, S-5 μm (Thermo Fisher Scientific).
(PDF)

S2 File Supporting information for S2 Table. (XLSX)

S1 Raw images. Raw western blot scans (PDF)
