# Supplementary figures and images for: A novel 3-acyl isoquinolin-1(2H)-one induces G2 phase arrest, apoptosis and GSDME-dependent pyroptosis in breast cancer
Source: PLoS One. 2022 May 12;17(5):e0268060. doi: 10.1371/journal.pone.0268060 (PMC9098002; doi:10.1371/journal.pone.0268060)

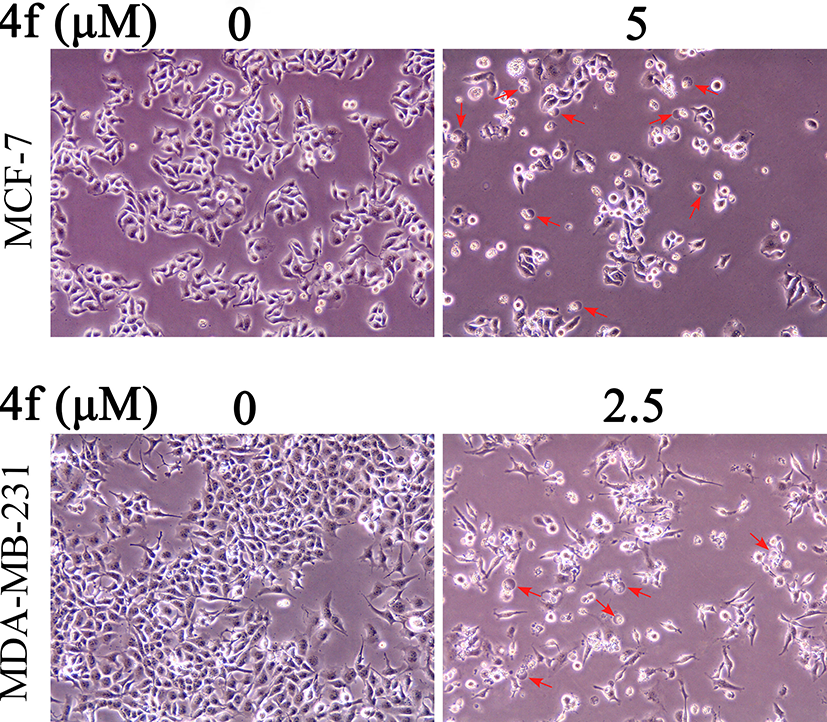

Supplement: S1 Fig — Representative microscopic images of MCF-7 and MDA-MB-231 cells after treated with 4f for 24 h with cell swelling. Red arrowheads indicate the characteristic balloon in the cell membrane, which are represent cells undergo pyroptosis. (TIF) [file pone.0268060.s001.tif]

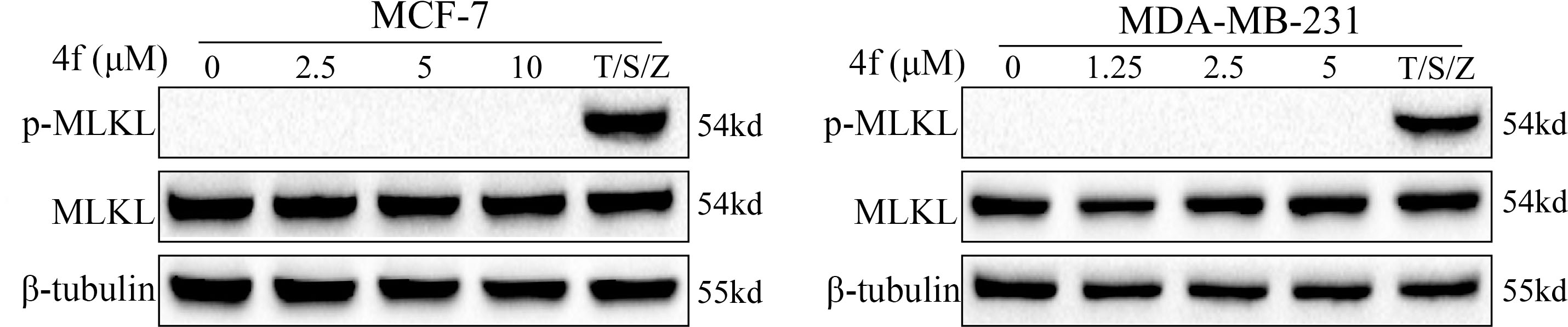

Supplement: S2 Fig — MCF-7 and MDA-MB-231 cells were treated with 4f or TSZ for 24 h, the total protein was extracted respectively, and then, the protein expression of p-MLKL and MLKL were analysized by western blotting analysis. TSZ is a combination of TNF-a (T, 20 ng/ml), Smac mimetic (S, 100 nM), and a pan-caspase inhibitor z-VAD-FMK (Z, 20 μM), which is used as necrosis inducer. (TIF) [file pone.0268060.s002.tif]
