# Supplementary material for: A novel 3-acyl isoquinolin-1(2H)-one induces G2 phase arrest, apoptosis and GSDME-dependent pyroptosis in breast cancer
Source: PLoS One. 2022 May 12;17(5):e0268060. doi: 10.1371/journal.pone.0268060 (PMC9098002; doi:10.1371/journal.pone.0268060)
Supplement: S1 Table — (DOCX) [file pone.0268060.s003.docx]

| Gene | Forward primer | Reverse primer |
| --- | --- | --- |
| CDK1 | AGCCGCCCTTTCCTCTTTCTTTC | CGGATTCACCAATCGGGTAGCC |
| β-tubulin | GCCACTGCCGCATCCTCTTC | AGCCTCAGGGCATCGGAACC |
