# Supplementary material for: A novel 3-acyl isoquinolin-1(2H)-one induces G2 phase arrest, apoptosis and GSDME-dependent pyroptosis in breast cancer
Source: PLoS One. 2022 May 12;17(5):e0268060. doi: 10.1371/journal.pone.0268060 (PMC9098002; doi:10.1371/journal.pone.0268060)
Supplement: S2 Table — Cells were treated with different concentrations of compound 4f for 48 h. IC50 values are the mean±SD (n = 3). (DOC) [file pone.0268060.s004.doc]

| Compounds | IC50 (μM) | |
| --- | --- | --- |
|  | MCF-7 | MDA-MB-231 |
| **4f** | 5.65±0.57 | 2.39±0.63 |
| 5-FU | 31.36±1.66 | 13.56±1.15 |
