# Supplementary material for: A novel 3-acyl isoquinolin-1(2H)-one induces G2 phase arrest, apoptosis and GSDME-dependent pyroptosis in breast cancer
Source: PLoS One. 2022 May 12;17(5):e0268060. doi: 10.1371/journal.pone.0268060 (PMC9098002; doi:10.1371/journal.pone.0268060)
Supplement: S1 File — HPLC conditions: Thermo Fisher C18 column HYPERSIL GOLD ODS, 250 x 4.6 mm I.D, S-5 μm (30:70 MeCN: H2O, 1.0 mL/min, 30°C, 254 nm); tr = 20.2 min, 99.3%. Instrument model: C18 chromatographic column HYPERSIL GOLD ODS, 250×4.6 mm I.D, S-5 μm (Thermo Fisher Scientific). (PDF) [file pone.0268060.s005.pdf]

S1 Fig. Copies of HPLC spectra of products 4f

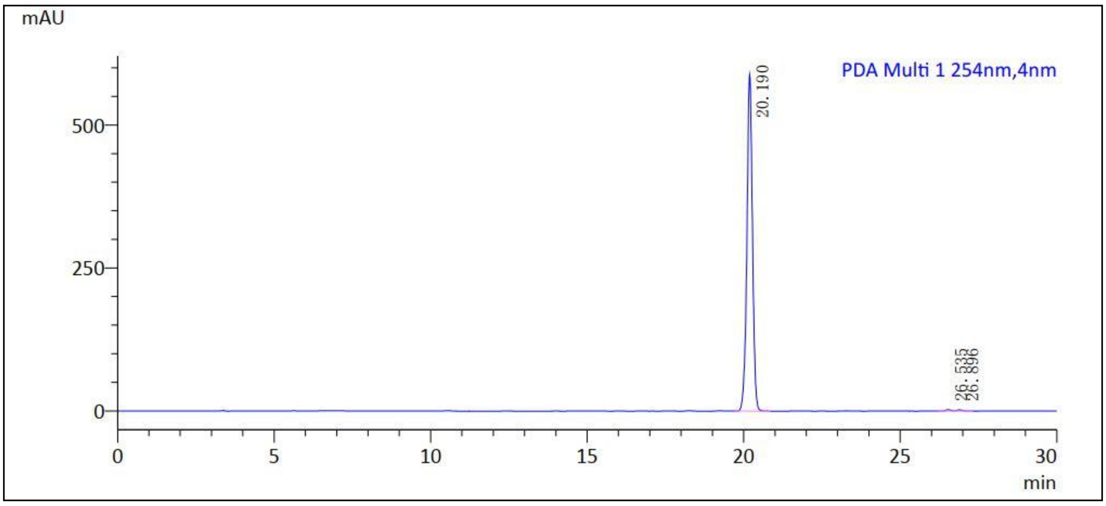

S1 Table. HPLC information of 4f

PDA Ch1
254nm

peak No	retention time	area	height	concentraton	area%
1	20.19	7230486	588081	99.253	99.25%
2	26.535	29146	2415	0.4	0.40%
3	26.896	25285	2284	0.347	0.35%
total		7284917	592780	100	100.00%

HPLC conditions: Thermo Fisher C_{18} column HYPERSIL GOLD ODS, 250 x 4.6 mm I.D, S-5 μm (30:70 MeCN: H_2O, 1.0 mL/min, 30 ^oC, 254 nm); tr = 20.2 min, 99.3%.

Instrument model: C18 chromatographic column HYPERSIL GOLD ODS，250×4.6 mm I.D，S-5 μm (Thermo Fisher Scientific)
