# Supplementary material for: A novel 3-acyl isoquinolin-1(2H)-one induces G2 phase arrest, apoptosis and GSDME-dependent pyroptosis in breast cancer
Source: PLoS One. 2022 May 12;17(5):e0268060. doi: 10.1371/journal.pone.0268060 (PMC9098002; doi:10.1371/journal.pone.0268060)
Supplement: S1 Raw images. Raw western blot scans — (PDF) [file pone.0268060.s007.pdf]

Fig 3E

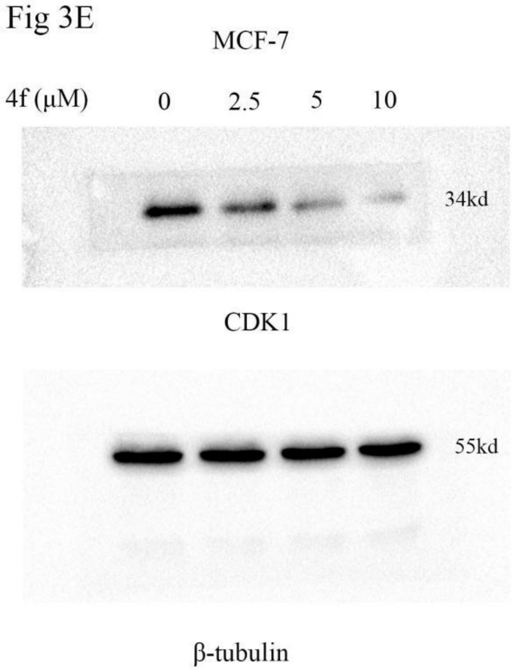
MCF-7
4f (μM)
0
2.5
5
10
34kd
CDK1
55kd
β-tubulin

Fig 3G

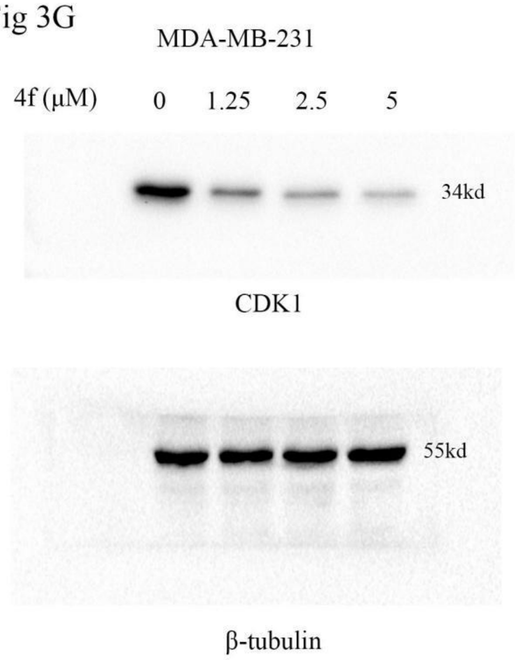
MDA-MB-231
4f (μM)
0
1.25
2.5
5
34kd
CDK1
55kd
β-tubulin

Fig 4C

MCF-7

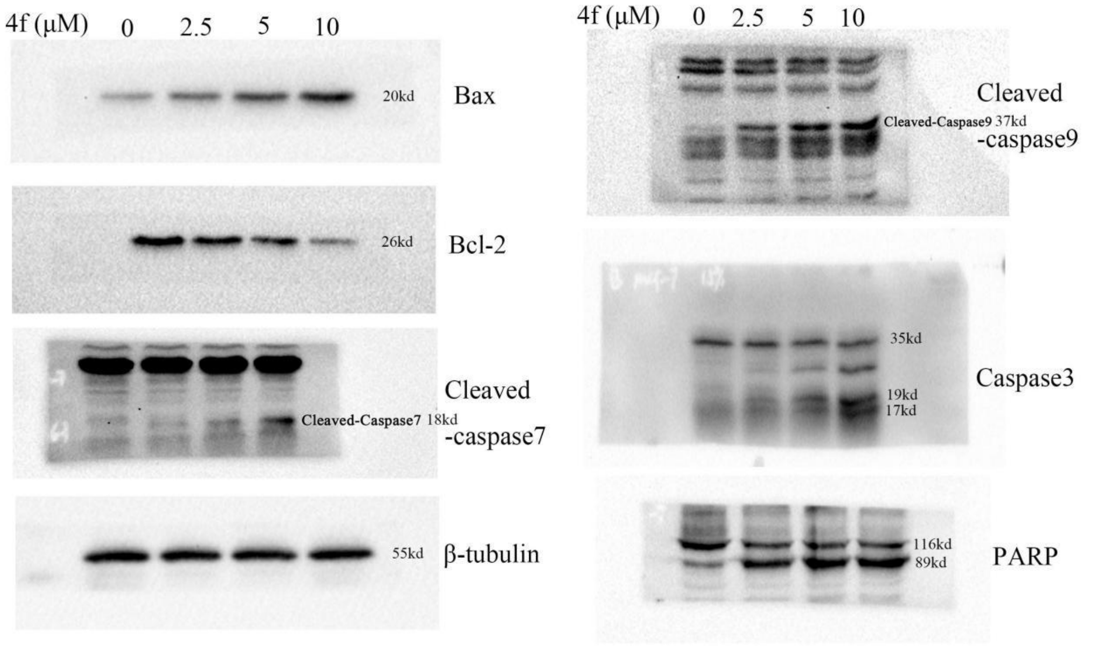

Fig 4D

MDA-MB-231

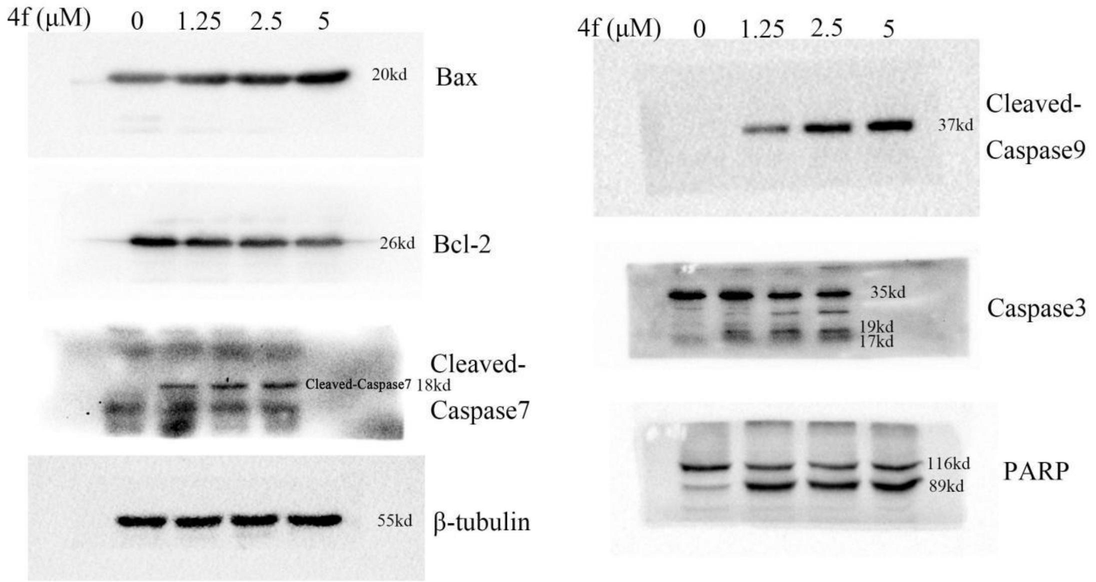

Fig 6A

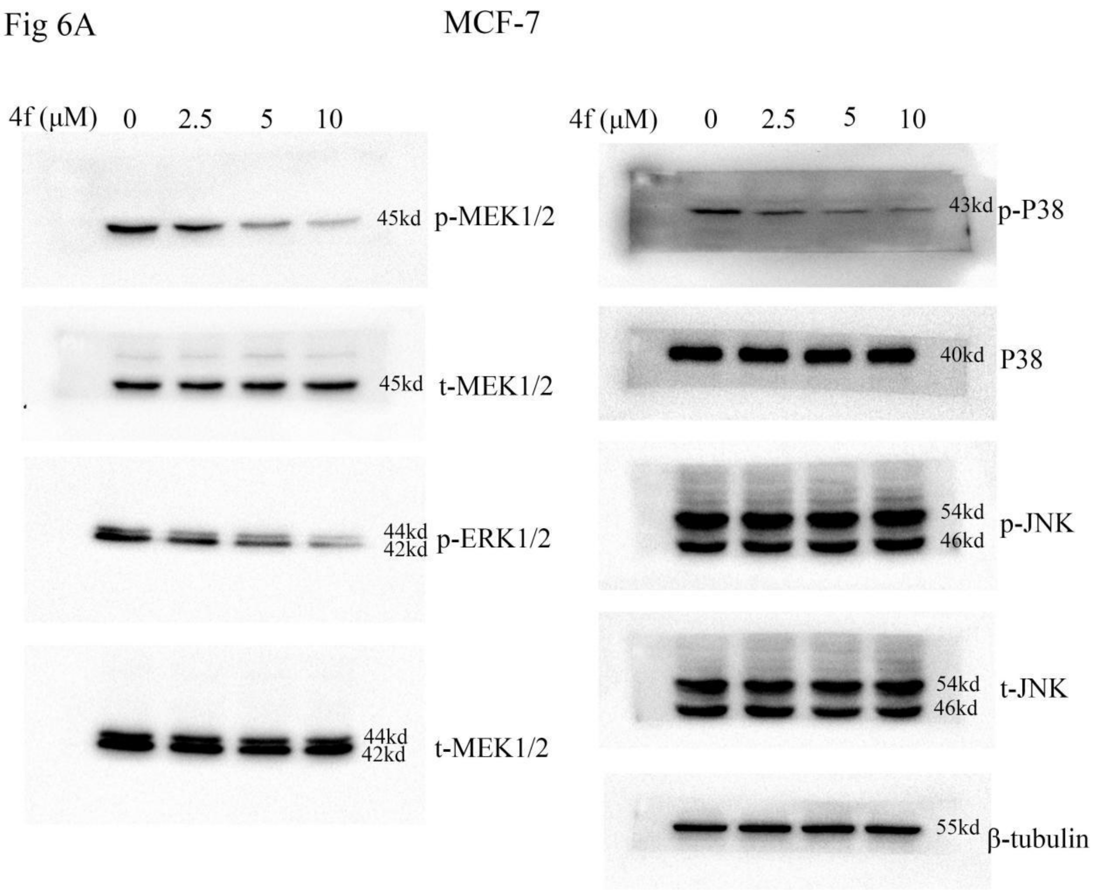
MCF-7
4f (μM) 0 2.5 5 10
45kd p-MEK1/2
45kd t-MEK1/2
44kd 42kd p-ERK1/2
44kd 42kd t-MEK1/2
4f (μM) 0 2.5 5 10
43kd p-P38
40kd P38
54kd 46kd p-JNK
54kd 46kd t-JNK
55kd β-tubulin

Fig 6B

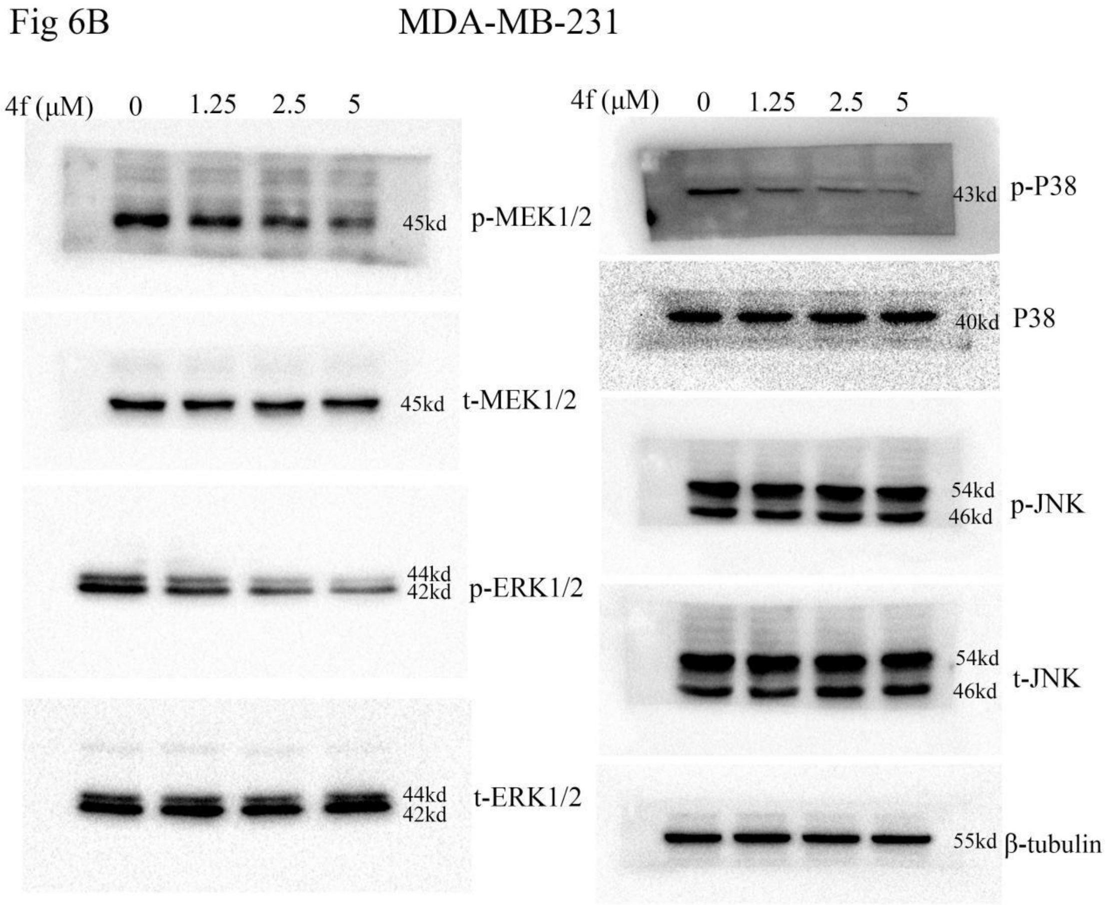
MDA-MB-231
4f (μM) 0 1.25 2.5 5
45kd p-MEK1/2
45kd t-MEK1/2
44kd
42kd p-ERK1/2
44kd
42kd t-ERK1/2
4f (μM) 0 1.25 2.5 5
43kd p-P38
40kd P38
54kd
46kd p-JNK
54kd
46kd t-JNK
55kd β-tubulin

Fig 7A MCF-7

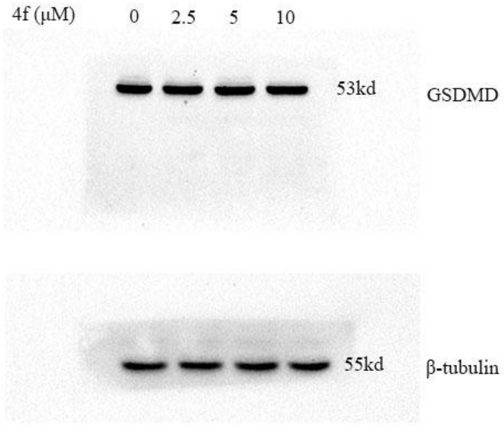

Fig 7B MDA-MB-231

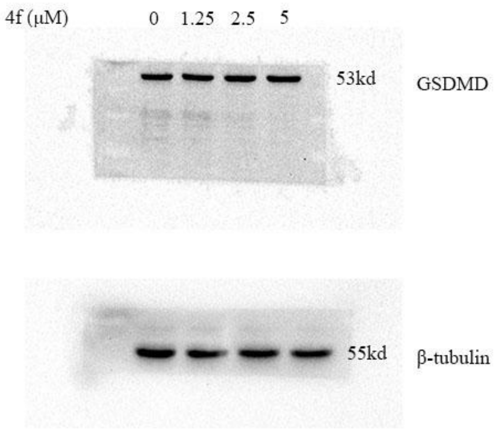

Fig 7C MCF-7

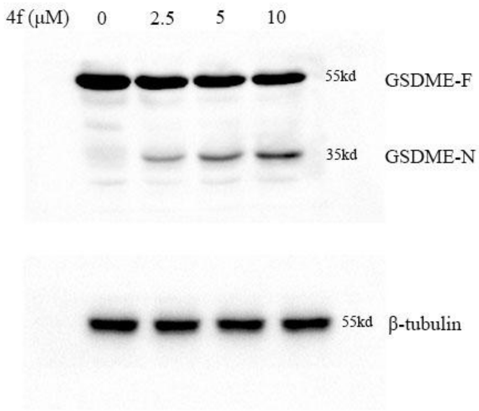

Fig 7D MDA-MB-231

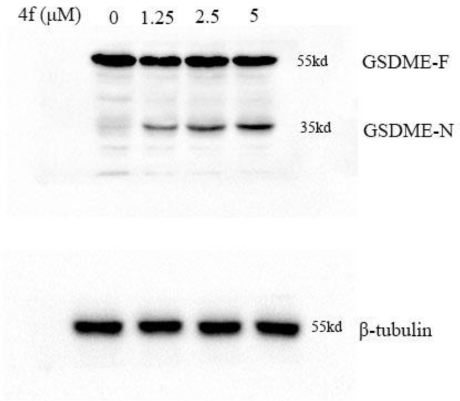

Fig 8A MCF-7

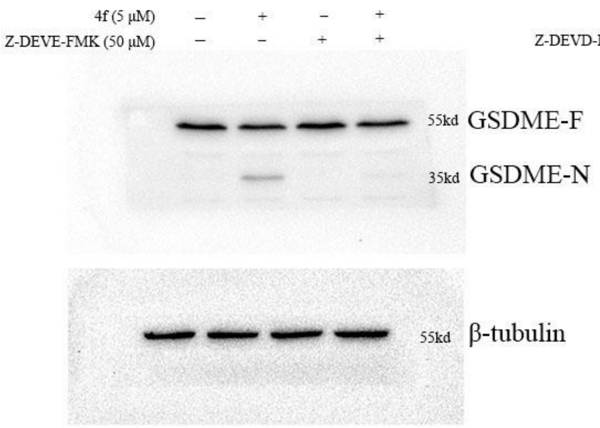

Fig 8D MDA-MB-231

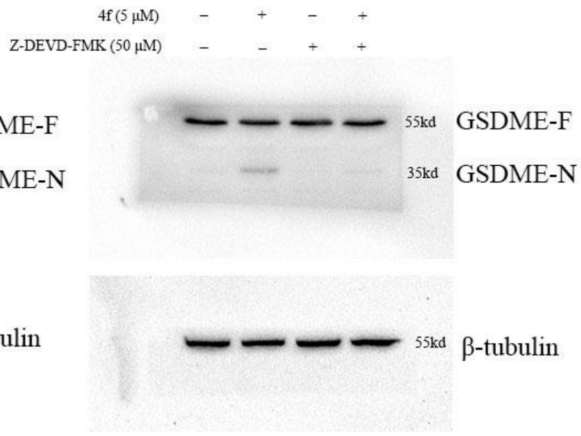

S2 Fig

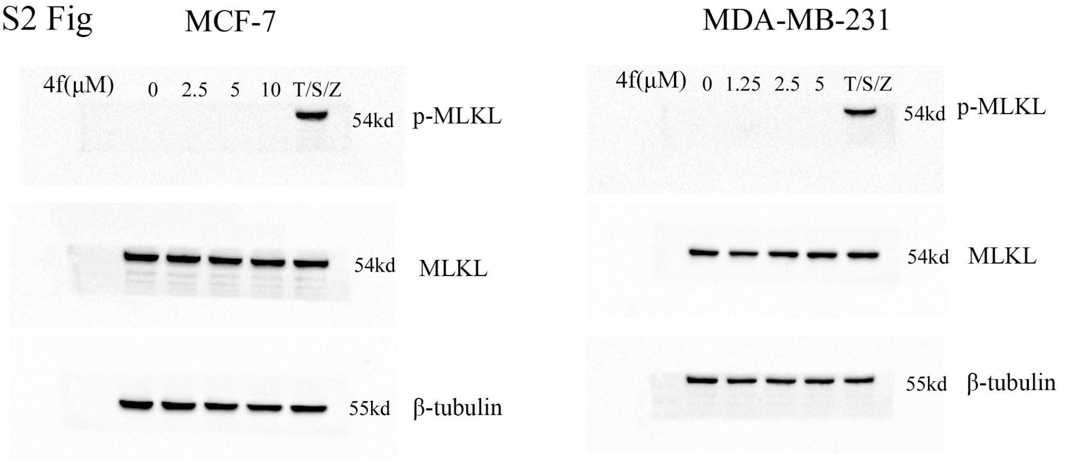

T/S/Z
T: TNF-α (20 ng/ml)
S: Smac mimetic (100 nM)
Z: Z-VAD-FMK (20 μM)
